# The perplexing pleura: Diagnosing tuberculosis pleural effusions in the era of drug resistance

**DOI:** 10.7196/AJTCCM.2019.v25i2.020

**Published:** 2019-07-31

**Authors:** Jane A Shaw, Elvis M Irusen

**Affiliations:** Division of Pulmonology, Stellenbosch University and Tygerberg Academic Hospital, Cape Town, South Africa

## Editorial

It is common knowledge that South Africa (SA) has a particularly high burden of tuberculosis (TB).^[1]^ This is reflected not only in the overall high incidence rate of TB, but also in the discrepancy between reported global and local rates of extrapulmonary TB (EPTB). Local data report that up to 42% of TB cases are EPTB, compared with the global rate of 15%.^[1,2]^ The local rate of pleural TB alone has been reported to be as high as 30%.^[3]^ While this is unsurprising in light of our high incidence of HIV coinfection, it does mean that we have to deal with a far higher burden of pleural TB than our colleagues in low-incidence regions. In fact, using international best estimates of TB burden, at a rate of 30%, SA would have had 96 600 cases of pleural TB in 2017. Yet the diagnosis of this common condition often still proves elusive. By its very nature, the TB pleural effusion is unlikely to have a high load of organisms. The rupture of a small subpleural caseous focus into the pleural space allows initial entry of the mycobacterium or its antigen, igniting an effusive T-helper type 1 celldriven immune response and a granulomatous pleuritis, which most commonly results in effective containment of the mycobacteria, and a particularly paucibacillary infection.^[4]^ Yields from traditional smear microscopy and cultures of pleural fluid are in general disappointingly low, even with the introduction of more effective culture media.^[5]^ For some time, the finding of a lymphocyte-predominant pleural exudate with a high level of pleural fluid adenosine deaminase (ADA) has been considered sufficient for the diagnosis of pleural TB within our high TB-incidence environment. With a positive predictive value of 98% for TB pleural effusion, this constellation of findings has been considered sufficient evidence to allow empiric anti-TB therapy.^[4,6]^ However, with our increasing awareness of the high rate of drug resistance among TB isolates, the role of empiric treatment has become questionable, and our diagnostic strategy has had to be revised. More patients now require pleural biopsy in an attempt to isolate the mycobacterium and identify its sensitivity profile. Alternative strategies have yielded some success – for example, mycobacterial culture of induced sputum samples in patients without any radiological evidence of pulmonary TB has a yield of up to 50% – but these are not always available.^[7]^ Although the polymerase chain reaction (PCR)-based Xpert MTB/RIF (Cepheid, USA) has made an impressive difference in the positive yield and turnaround times for the diagnosis of pulmonary TB, particularly in populations with smear-negative disease and rifampicin resistance, its performance in pleural fluid has been somewhat disappointing.^[8]^ There just do not seem to be enough mycobacteria in the average TB pleural effusion to reach detectable levels. The real trouble is not in those who have evidence of pulmonary TB on chest radiograph (in whom the investigation with the highest yield would be a sputum Xpert and culture), but those who do not. Certain factors have been identified that make the mycobacterial load higher, and consequently increase yields in smear microscopy and culture of pleural fluid, including pleural fluid neutrophilia, loculated effusions and HIV coinfection.^[4]^ Theoretically, one would expect that these factors, or other identifiable population characteristics, would translate into an increased detection rate for Xpert on pleural fluid as well. In a setting where cost constraints exist, it would be helpful to have an idea of which patients are likely to yield a positive Xpert on pleural fluid prior to performing the pleural aspiration, to avoid unnecessary costs. In this issue of the journal, Makambwa *et al*. ^[9]^ examine this very question. They performed an analysis of a cohort of 49 patients with proven TB pleural effusions, trying to identify characteristics within these cases that predicted Xpert (specifically the Xpert Ultra cartridge) positivity in pleural fluid. Of these 49 patients, 38 had no radiological evidence of pulmonary TB; in other words, they had isolated pleural TB. Within the cohort as a whole, the only factor that was associated with a positive Xpert with statistical significance was the presence of radiological evidence of active pulmonary TB – a population in whom the TB is probably best diagnosed by other means than pleural fluid analysis. In a study with a small sample size, it is unhelpful to fixate on p-values, and this study has a few findings worthy of comment despite the lack of statistical significance. Firstly, the baseline characteristics of the pleural fluid analysis are in keeping with the picture described above, which is known to have a high positive predictive value for pleural TB, that is, a high fluid protein, ADA and lymphocyte count. Interestingly, the mean values for protein, ADA and lymphocytes were all significantly higher in the group that was Xpert-positive. A curious feature which, when combined with the fact that only 37% (n=13/35) of the cases that were culture-positive on pleural fluid were also Xpert-positive, emphasises again that these secondary indicators of pleural TB might be more reliable than direct methods of mycobacterial detection for simply making the diagnosis. Secondly, the association of Xpert positivity with pleural effusion size is the first of its kind that we have seen. Notably, the incidence of small, moderate and large effusions in this study roughly correlates with other reports.^[10]^

The message from this study for those of us in clinical practice seems to be that as a diagnostic tool for pleural TB, the Xpert leaves much to be desired, and still does not match the sensitivity of basic pleural fluid analysis. Practically speaking, when there are signs on a chest radiograph of active pulmonary TB, one should still perform sputum Xpert and culture for the highest diagnostic yield, whether or not there is a pleural effusion. In patients who have isolated effusions, a diagnosis can be inferred by the presence of a lymphocyte-predominant exudate with a high ADA, but caution is advised: drug resistance is a real danger and for this reason, even with the low yield of Xpert in this setting, its use might still be justified.
